# Protein-Level Analysis of Differential Response to Chemotherapy in Triple-Negative Breast Cancer Identifies CYP1B1 as a Biomarker for Chemotherapy Resistance

**DOI:** 10.1158/2767-9764.CRC-25-0034

**Published:** 2025-07-01

**Authors:** F. Scott Heinemann, Paul D. Gershon

**Affiliations:** 1 Department of Pathology, Hoag Memorial Hospital Presbyterian, Newport Beach, California.; 2 Department of Molecular Biology and Biochemistry, University of California, Irvine, California.

## Abstract

Resistance to chemotherapy is a critical challenge in triple-negative breast cancer (TNBC). In this study, the proteomes of pretreatment core biopsy samples from 16 patients with TNBC with differential response to neoadjuvant chemotherapy (NAC) were analyzed by nanoLC/MS-MS to identify biomarkers of intrinsic chemotherapy resistance. This led to the identification of cytochrome P450, family 1, subfamily B, polypeptide 1 (CYP1B1) and 71 additional proteins as significantly more abundant in chemoresistant than chemosensitive TNBC. IHC analysis of 80 TNBC samples confirmed an association between elevated tumor cell CYP1B1 and residual cancer burden class 2/3 disease after NAC in T cell–excluded (TCE) TNBC (*P* < 0.01) but not in T cell–infiltrated (TCI) TNBC. The frequency of complete pathologic response in TCE-TNBC with elevated CYP1B1 was 18% versus 56% in TCE-TNBC with low CYP1B1 and 75% in TCI-TNBC. Retrospective review of the chemotherapy regimens suggested that TCE-TNBC with elevated CYP1B1 was particularly resistant to doxorubicin/cyclophosphamide. This study is the first to associate resistance to NAC in TNBC with elevated CYP1B1.

**Significance:**

This retrospective analysis of pretreatment TNBC core biopsies found that elevation of CYP1B1, a drug-metabolizing enzyme in tumor cells, was associated with resistance to NAC in patients with TNBC treated initially with doxorubicin. If confirmed, this pattern of chemotherapy resistance could guide future clinical trials.

## Introduction

Triple-negative breast cancer (TNBC) is a clinically, histologically, and molecularly heterogeneous disease (1–3). Although this suggests a need for therapies tailored to specific TNBC subgroups, no targeted therapies have yet been developed. Preoperative neoadjuvant chemotherapy (NAC) is the standard treatment for early-stage high-risk TNBC, but response to NAC is variable and unpredictable. Patients who have no residual cancer after NAC have a significantly improved disease-free survival compared with patients who have residual disease (4). Calculation of the residual cancer burden (RCB) predicts the risk level for recurrence with greater accuracy than the presence or absence of residual disease (5). Furthermore, patients with class 2 or class 3 RCB are at significantly higher long-term risk of a fatal outcome in comparison with patients with no residual invasive carcinoma (RCB class 0) or RCB class 1 disease (6, 7). Thus, NAC distinguishes patients with intrinsically chemosensitive tumors, who have an excellent prognosis after chemotherapy and surgery, from those with chemoresistant tumors who remain at high risk after therapy. Biomarkers that predict chemotherapy resistance could help guide future clinical trials.

RNA expression profiling has dominated the search for breast cancer biomarkers for the past quarter century. These efforts have led to multigene assays that predict breast cancer outcomes (8, 9) as well as genomic classifications of breast cancer (10) and TNBC (1, 11). Consistently, the evaluation of global DNA methylation patterns in TNBC (12) has supported the hypothesis that TNBC pathobiology is driven by epigenomic dysregulation (13). Protein-based efforts toward biomarker discovery have lagged nucleic acid research but have more recently benefited from advances in mass spectrometry (MS)–based approaches to protein analysis (14, 15). One advance critical for the study of formalin-fixed, paraffin-embedded (FFPE) tissue has been the finding that “antigen retrieval” procedures developed for the augmentation of IHC staining (16) facilitated the solubilization of proteins in FFPE tissue (17) and the preparation of enzymatic protein digests suitable for LC/MS-MS (18). Tumor cell–enriched proteomes of FFPE breast cancer tissue have been obtained for nanoLC/MS-MS analysis through the use of laser capture microdissection (19), manual macrodissection (20), and histologically targeted *in situ* digestion (21).

The aim of this investigation was to identify differentially abundant proteins in chemoresistant TNBC by nanoLC/MS-MS and to validate selected biomarkers by IHC. Histologically targeted *in situ* digestion was used to acquire tumor cell–enriched proteomes. Special histologic subtypes of TNBC were excluded from the discovery cohort. From the 72 proteins identified by MS as potential biomarkers for chemoresistance, cytochrome P450, family 1, subfamily B, polypeptide 1 (CYP1B1) was selected for validation by IHC.

## Materials and Methods

### Tumor samples

Patients whose diagnostic core biopsy and surgical resection were performed at Hoag Memorial Hospital Presbyterian were eligible for the study. Tumor registry data and pathology records were reviewed to identify patients with TNBC who had undergone NAC and were clinical stage cT1c or greater prior to treatment and pathologic stage ypT0 or ypT1b or greater after treatment. TNBC was defined as a core biopsy result that was estrogen receptor–negative, progesterone receptor–negative, and HER2-negative based on the American Society of Clinical Oncology/College of American Pathologists recommendations for interpretation of estrogen receptor, progesterone receptor, and HER2 IHC stains and FISH assays for HER2 (22, 23). These criteria were met by 80 patients treated between 2015 and 2022, including 38 patients classified as ypT0 and 42 patients with residual disease most of whom were classified as either ypT1c or ypT2. Subsequent to this selection process, the resection slides of patients with residual disease were reviewed and the RCB class was determined using the RCB calculator (http://www3.mdanderson.org/app/medcalc/index.cfm?pagename=jsconvert3, accessed April 20, 2025). Based on final pathologic review, the 80 patient cohort contained 48 and 32 core biopsies associated with an RCB class 0/1 outcome and an RCB class 2/3 outcome, respectively. A subset (*N* = 16) of tumor samples was selected for MS analysis as follows: All MS cohort samples were histologic grade 3 invasive ductal carcinoma of no special type with elevated (>60%) Ki67 proliferation index. Pleomorphic lobular TNBC, metaplastic TNBC, and apocrine TNBC were excluded from the MS cohort. Tumors with stromal lymphocyte (sTIL) density >50% were excluded from the MS cohort. Eight MS cohort samples had an RCB class 0 outcome, seven had an RCB class 2 outcome, and one had an RCB class 3 outcome. All MS cohort samples had at least one tissue core with at least 3 mm of tumor tissue in which the proportion of malignant cells (tumor cellularity) was at least 40%.

### nanoLC/MS-MS

Sample preparation for MS by *in situ* digestion was as previously described (21), except that quadruplicate samples were employed in place of triplicates. In brief, 4-micron sections were mounted on adhesive slides and then prepared for digestion by baking, deparaffinization, heating in antigen retrieval buffer, rinsing, and drying. Regions of high tumor cellularity ranging from 3 to 6 mm in length were localized on the unstained slides by comparison with a hematoxylin and eosin (H&E)–stained serial section and outlined with ink. Lys-C digestion mix was applied to the target area for 40 minutes and then collected with five volumes of 50 mmol/L triethylammonium bicarbonate buffer, followed by addition of trypsin mix, incubation at room temperature, and then storage at −80°C. After supplementation with formic acid to 2% final concentration, peptides were desalted, dried, and redissolved in 0.1% formic acid in water as described (21). The above sample quadruplicates were prepared from two patient cohorts (C1 and C2) selected 5 months apart. The two cohorts each comprised eight TNBC samples [four pathologic complete response positive (pCR+) and four pathologic complete response negative (pCR−)]. As a time-dependent “drift” control for MS, the analysis of one pCR+ tumor in C1 was repeated utilizing serial paraffin sections that had been stored at room temperature for 5 months. Data from the two cohorts were combined in the final analysis. MS was performed as described in ref. (21).

### Analysis of nanoLC/MS-MS data

MS raw files were imported to MaxQuant (24). The Andromeda search engine in the MaxQuant software suite was utilized as described (21). The “Protein groups.txt” output from MaxQuant was uploaded to “Perseus” (25) in which matrix was filtered to exclude common contaminants, hits to the reverse database, and proteins identified only via modified peptides. Sample groupings were defined by either the patient identifier or pCR−/pCR+ response group. Quant values were logarithmized (base 2) after which the dataset was filtered for proteins represented by at least 20 valid quant values in each pCR group. Missing quant values were replaced with random values imputed from a normal theoretical intensity distribution representing the left (low intensity) end of the intensity histogram using default values for Gaussian width and down shift. Imputation was performed three times, and the resulting three sets of intensity histograms were visually inspected to ensure only a minimal contribution of imputed values. The following was done for each of the three resulting datasets: for each sample, median log(2) intensity over all proteins was subtracted from the log(2) intensity for each individual protein as a normalization step. The resulting log(2) quant values were subjected to a two-sided *t* test on the basis of pCR grouping (above), with 250 random reassignments of samples to the two response groups leading to a *P* value. Volcano plots of *P* value (Y) versus log(2) differential abundance show a significance line defined by a 5% FDR and an S0 parameter of 0.1. Volcano-significant protein sets arising from each of the three distinct imputations were exported and the set of volcano-significant proteins was compared using in-house code and found to be identical. The dataset from imptation1 was further subjected to multi-way ANOVA analysis on the basis of either the patient or response group, and proteins showing ANOVA-significant (with a permutation-based FDR threshold of 5%) differential abundance were subjected to *Z* score normalization and then visualized by heatmap representation with Euclidian distance/average linkage hierarchical clustering.

### Histologic and IHC analysis; TNBC immunotype criteria

sTIL density was determined in H&E-stained slides by an established method (26). Intratumoral T cells (ITC), defined as T cells in contact with tumor cells, were enumerated in sections double stained for CD3 (brown) and pankeratin (red). The ITC density of each tumor sample was determined as described (21), except that ITC density was determined in five 20X (1.1 mm) fields (as opposed to one, in the prior study), and these values were averaged to yield a mean ITC density for each sample. TNBC immunotypes were defined by previously published criteria (21) with modification as follows: TCD-TNBC were defined by <10% sTIL density; TCR-TNBC were defined by >10% sTIL density and <15% ITC density; TCI-TNBC were defined by >15% ITC density. All IHC staining was performed using a BOND III automated IHC stainer (Leica Biosystems) as previously described (21). IHC staining for CD3 and pankeratin was performed according to procedures developed in the Hoag Hospital Pathology laboratory without modification as previously described (21). CYP1B1 stains were optimized as described (21). Primary antibodies used for IHC in this study were CD3 (NCL-L-CD3-565; Novocastra, RRID: AB_563541), pankeratin (NCL-L-C11; Novocastra), cytochrome P4501B1, polyclonal (dilution 1:100, cat. #18505, Proteintech), and CYP1B1 monoclonal antibody (clone #EPR14972, dilution 1:2000, cat. #ab185954, Abcam, RRID: AB_2894869). Antigen retrieval for CYP1B1 staining was done on the BOND III instrument using Leica ER2 antigen retrieval buffer for 20 minutes. The antibody incubation time for CYP1B1 antibodies was 60 minutes. CYP1B1 histoscores were based on the proportion of tumor cells stained, the stain intensity, and the subcellular distribution of staining. Cytoplasmic staining in viable neoplastic cells was quantized by the weighted histoscore as follows: (% of unstained tumor cells × 0) + (% of weakly stained tumor cells × 1) + (% of moderately stained tumor cells × 2) + (% of strongly stained tumor cells × 3) to produce values in the range 0 to 300.

### Chemotherapy data

Tumor registry data and patient charts were reviewed to determine the chemotherapy protocols used.

### Statistical methods for patient subgroup analysis

The *χ*^2^ statistic was used to evaluate the frequency of response groups (pCR+ vs. pCR− and RCB class 0/1 vs. RCB class 2/3) in TNBC subsets defined by sTIL density, ITC density, and CYP1B1 histoscore. A Student *t* test (two-tailed, type 3) was used to compare the mean CYP1B1 histoscores of response subgroups.

### Institutional Review Board review

The study reported herein was approved by the WIRB-Copernicus Group Institutional Review Board (protocol number 100-22-CA-E) and conducted according to the principles expressed in the Declaration of Helsinki.

### Data availability

The data generated in this study are available upon request from the corresponding author.

## Results

### MS identifies CYP1B1 as a biomarker of chemoresistance in TNBC

The TNBC biopsies in the MS cohort were selected to represent a single histologic tumor type, namely grade 3 invasive ductal carcinoma of no special type with ≥60% Ki67 proliferation index. Regions of high tumor cellularity were targeted for digestion and nanoLC/MS-MS analysis in order to obtain tumor cell–enriched proteomes. The median tumor cellularity and sTIL density of the targeted foci were 72.5% and 60% and 17.5% and 15% in the pCR+ and pCR− subsets of the MS cohort, respectively (Supplementary Table S1). As reported previously (21), cellular material in the targeted regions was removed completely by the *in situ* digestion procedure (Supplementary Fig. S1). Notwithstanding their uniform histologic classification, the targeted foci had unique morphologic features (Supplementary Fig. S2).

**Figure 1:**
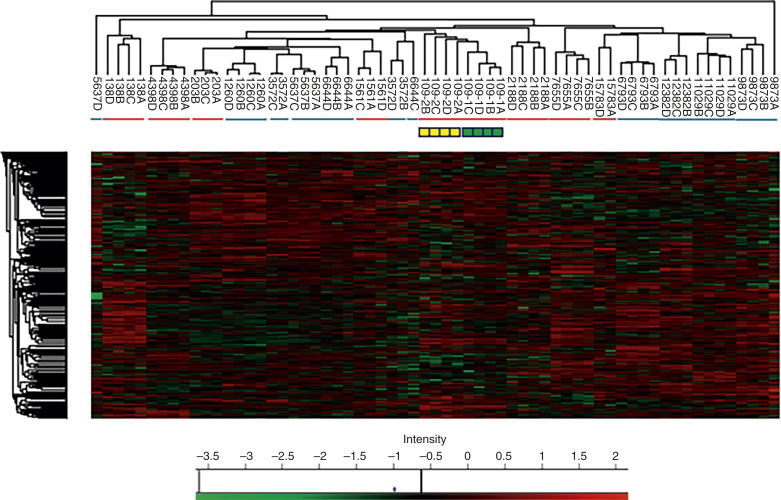
Unsupervised hierarchical clustering, with heatmap, for 1,622 proteins identified as significantly differentially abundant in any patient grouping among 16 patients with TNBC in two clinical response groups. Heatmap values represent log(2) *Z*-scored LFQ intensity values (for individual proteins minus the median value for all proteins from the same sample). Heatmap red and green coloration represents overabundant, under-abundant proteins, respectively. Colored bars under the vertical (column) sample clusters represent the response phenotype (red, pCR+; blue, pCR−). For samples 109, 263, 1,260, 1,561, 3,572, 4,398, 5,637, and 6,694, proteomes A and C and B and D represent serial sections from core 1 and core 2, respectively, of a single biopsy. For other patients, proteomes A to D represent serial quadruplicate sections from a single core. Yellow/green boxes beneath patient 109 show an MS drift control: A core biopsy for which digestion/sample processing and nanoLC/MS-MS were repeated on stored serial sections after a 5-month interval (yellow and green – repeat and initial, respectively). Horizontal (row) clusters represent protein groupings.

Pilot scale (“single-shot”) MS runs of quadruplicate serial sections from the 16 sample discovery cohort led to the identification of 3,704 proteins in at least one tumor sample, of which after data filtering and processing (“Materials and Methods”), 1,622 were ANOVA significant for differential abundance in at least one patient with respect to other patients (Fig. 1). Proteomes of serial quadruplicate sections of samples predominantly formed the leaf nodes of hierarchical sample clusters (Fig. 1), demonstrating the reproducibility of quantitative proteomes obtained by this approach. Of note, the samples were analyzed in two cohorts (C1 and C2), with cases selected and digests prepared 5 months apart. The entire process of digestion, desalting, and nanoLC/MS-MS was repeated for one C1 sample (#109) using serial paraffin sections that had been stored for 5 months. As shown, the proteomes of the initial quadruplicate digests and repeat digests (109-1A–1D and 109-2A–2D, respectively) co-clustered (Fig. 1, green and yellow boxes, respectively). This finding further validated the reproducibility of proteomes obtained by *in situ* digestion and suggested that FFPE tissue proteomes were stable in storage as thin paraffin sections as recently reported (27).

**Figure 2:**
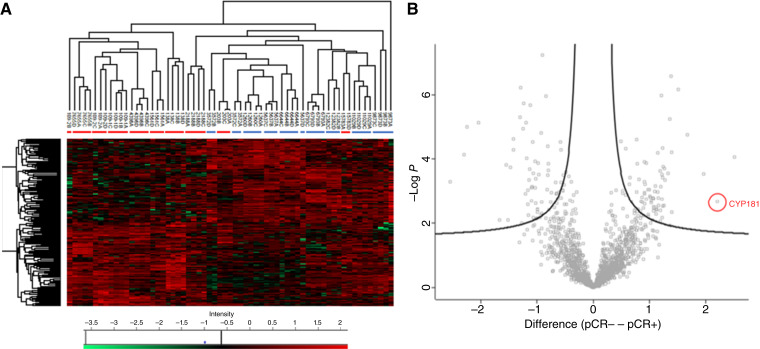
Differentially abundant proteins in pCR+ TNBC and pCR− TNBC. **A,** Unsupervised hierarchical clustering, with heatmap, for 160 proteins identified as significantly differentially abundant in pCR+ and pCR− TNBC. Details are as under Fig. 1. **B,** Volcano scatter plot showing protein differential abundance in pCR+ and pCR− TNBC. Each point represents an individual protein. A response group–based two-sided *t* test was performed for each of the proteins in the dataset after filtering steps, imputation, and mean subtraction (“Materials and Methods”). *X*-axis: difference between mean log(2) LFQ intensity for the two clinical response groups. *Y*-axis: −log(10) *t* test *P* value for differential abundance. Points representing proteins determined to be significantly overabundant in pCR+ and pCR− TNBC are shown to the left and right, respectively, of the volcano plot. The point representing CYP1B1 (the second most overabundant protein detected in pCR− TNBC) is ringed. The full list of 135 proteins identified by volcano analysis are listed in Supplementary Table S2.

No clear segregation of the clinical phenotypes was evident in the patient-grouping dendrogram (Fig. 1; pCR+ and pCR− patients indicated by red and blue bars, respectively), indicating that differential protein abundance between histologically similar TNBC was largely unrelated to the chemotherapy response phenotype. Consistent with this, just 160 proteins (∼10% of the number differing between patients) were significantly differentially abundant between the two response groups (Fig. 2A). Volcano analysis of the dataset identified 63 and 72 proteins that were significantly under-abundant and overabundant, respectively, in pCR− versus pCR+ samples (Fig 2B). The 135 proteins identified by volcano analysis are listed in Supplementary Table S2. Within the 72-protein group, literature review indicated several that are implicated in chemotherapy resistance including HLA-G (the most overabundant protein in the whole set; ref. 28), CYP1B1 (29), SLC7A5 (the second most overabundant; ref. 30), and CHD4 (31). Because of its well-established metabolic function (32, 33) and potential for inhibitor-based modulation (34, 35), CYP1B1 was selected for IHC analysis.

### IHC validates CYP1B1 as a biomarker of chemotherapy resistance in TNBC

**Figure 3:**
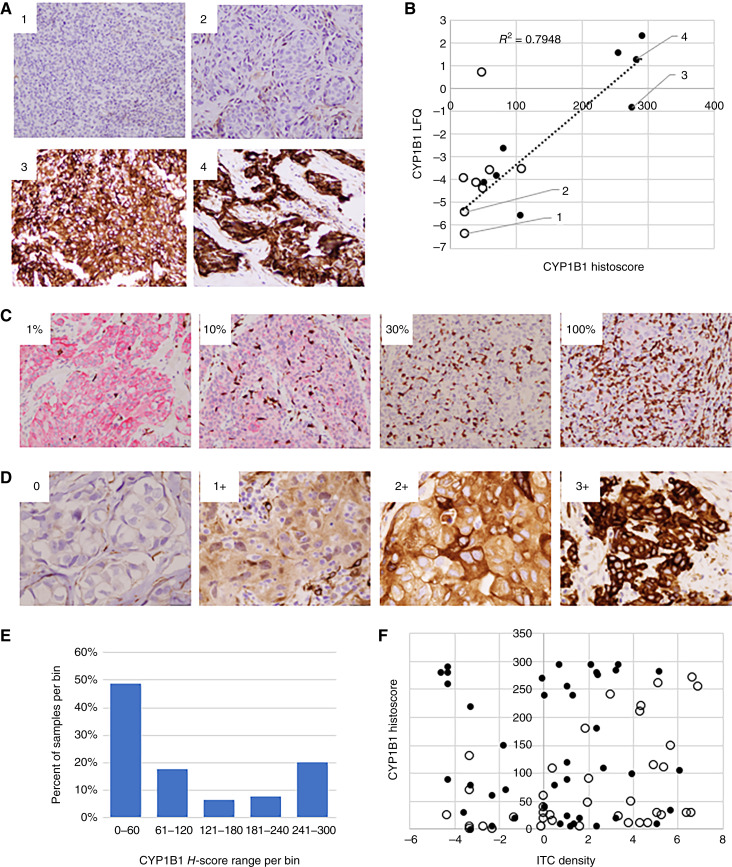
Validation of CYP1B1 as a biomarker of chemotherapy resistance in TNBC. **A,** CYP1B1 staining with ab185954 of two pCR+ MS cohort patients (1, 2) and two pCR− MS cohort patients (3, 4). Images were obtained with a 20X objective. **B,** CYP1B1 histoscores (*x*-axis) vs. CYP1B1 quadruplicate mean LFQ values (*y*-axis) for the 16 patients in the MS cohort. White and black points represent pCR+ and pCR− patients, respectively. **C,** Multikeratin/CD3 double stains of representative TNBC with distinct ITC densities. Tumor cells and T cells are stained red and brown, respectively. Estimated ITC densities in the respective images are shown. Images were obtained with a 20X objective. **D,** CYP1B1 IHC stains of TNBC with negative (0), weak (1+), moderate (2+), and strong (3+) staining intensity in tumor cell cytoplasm. Images were obtained with a 40X objective. **E,** Histogram in which bins represent CYP1B1 histoscore groups (*x*-axis) versus percentage patient samples in each bin (*y*-axis). **F,** Distribution of log_2_ ITC density (*x*-axis) vs. CYP1B1 histoscore (*y*-axis) in 80 patients with TNBC. Patients associated with pCR+ and pCR− outcomes are colored white and black, respectively.

To further investigate the association between elevated CYP1B1 in TNBC and resistance to chemotherapy, IHC assays for CYP1B1 were developed. Two CYP1B1 antibodies were evaluated, a monoclonal (Abcam, cat. #ab185954) and a polyclonal (Proteintech, cat. #18505). Among the 16 TNBC core biopsy samples subjected to nanoLC/MS-MS, both antibodies produced cytoplasmic staining in tumor cells which correlated well with CYP1B1 abundance values determined by MS via label free quantification (LFQ). Although the polyclonal antibody produced less background staining, the monoclonal antibody produced optimal staining at a lower concentration and was therefore selected to stain the remaining IHC cohort sections. Figure 3A shows ab185954 IHC stain results with sections from four representative MS cohort samples, two each representing low and high CYP1B1 LFQ abundance (images labeled 1 and 2, and 3 and 4, respectively). IHC and MS-LFQ quantitations of CYP1B1 among the 16 patients with TNBC in the MS cohort were strongly correlated (*r*^2^ = 0.79; Fig. 3B), with a median CYP1B1 histoscore of 65. The CYP1B1 histoscore for seven (of eight) pCR+ MS cohort TNBC was below the median whereas seven (of eight) pCR− MS cohort TNBC had an above-median histoscore. Furthermore, the CYP1B1 histoscore for four pCR− MS cohort TNBC was >240, whereas the highest CYP1B1 histoscore in the pCR+ MS cohort was 108 (Fig. 3B).

To further validate the association between elevated CYP1B1 in TNBC tumor cells and chemoresistance, CYP1B1 histoscores were determined in 64 additional pretreatment core biopsy samples from patients treated with NAC and classified upon resection as pCR+ or pCR−. To assess the interaction of CYP1B1 with the tumor microenvironment (TME), sTIL density and ITC density were also quantified. sTIL density was quantified in H&E-stained sections by an established method (26). ITC density was determined in sections double stained for CD3 (brown) and multikeratin (red) to localize T cells in relation to epithelial cells (Fig. 3C) as previously described (21). As seen in the MS cohort patients, the ab185954 IHC stain intensity in tumor cells was variable (Fig. 3D). When tumor cell CYP1B1 was quantified by the weighted histoscore method, the distribution of CYP1B1 histoscores across the 80-sample TNBC cohort was bimodal (Fig. 3E). Approximately 50% of tumors were CYP1B1 negative or weakly positive and 20% of tumors had histoscores >240 (Fig. 3E). Relatively few tumors had an intermediate CYP1B1 histoscore. The median CYP1B1 histoscore was 70 in the 80-sample TNBC cohort.

Tumor stage after NAC was significantly associated with both ITC density and CYP1B1 histoscore. A total of 75% of patients (15 of 20) with T cell–infiltrated (TCI) TNBC, defined as ITC density >15%, were from the pCR+ response group (Fig. 3F; Table 1). By contrast, only 38% of patients (23 of 60) with T cell–excluded (TCE) TNBC, defined as ITC density <15%, were pCR+ (Fig. 3F; Table 1). Across the entire cohort, 61% of patients (25 of 41) with a below median CYP1B1 histoscore were pCR+, whereas 33% (13 of 39) of patients with above median CYP1B1 were pCR+ (*P* < 0.025, *χ*^2^ test). Interestingly, the association between CYP1B1 and post-NAC tumor stage was driven entirely by the TCE-TNBC subset (Fig. 3F). Within the TCI-TNBC subset, eight of 11 tumors with above median CYP1B1 and seven of nine tumors with below median CYP1B1 were pCR+ (Fig. 3F). However, within the TCE-TNBC subset, only 18% (five of 28) of TNBC with above median CYP1B1 were pCR+, whereas 56% (18 of 32) of TCE-TNBC with low CYP1B1 were pCR+ (*P* < 0.01; Table 1).

**Table 1:** Frequency of pCR in relation to the TME in TNBC (*N* = 80) and in relation to CYP1B1 histoscore in TCE-TNBC (*N* = 60)

TNBC subgroup	*N*	pCR (*N*)	pCR (%)	*P*
TCE-TNBC	60	23	38%	<0.01
TCI-TNBC	20	15	75%	
TCE-TNBC, CYP1B1 <75	32	18	56%	<0.01
TCE-TNBC, CYP1B1 >75	28	5	18%	

Abbreviations: CYP1B1 <75, CYP1B1 histoscore <75; CYP1B1 >75, CYP1B1 histoscore >75; pCR, pathologic complete response; TCE, T cell excluded; TCI, T cell infiltrated; TME, tumor microenvironment.

*P* < 0.01 and *X*^2^ = 8.0869 for TCE-TNBC vs. TCI-TNBC. *P* < 0.01 and *X*^2^ = 9.317 for TCE-TNBC, CYP1B1 <75 vs. TCE-TNBC, CYP1B1 >75.

### Elevated CYP1B1 is associated with high-risk RCB in TNBC

**Figure 4:**
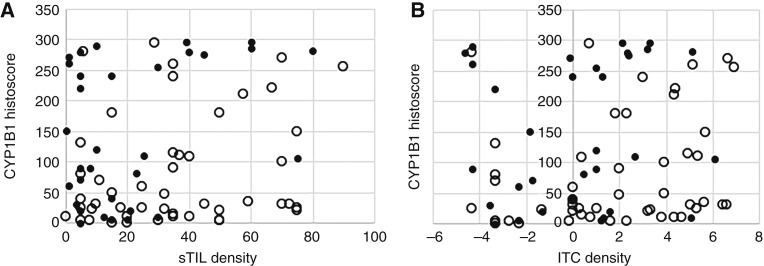
CYP1B1 histoscore vs. TME metrics in 80 patients with TNBC in two chemotherapy response groups defined by RCB. **A,** Scatterplot of sTIL density (*x*-axis) vs. CYP1B1 histoscore (*y*-axis) in 80 patients with TNBC. White and black points represent patients with TNBC with RCB class 0/1 and RCB class 2/3 outcomes, respectively. **B,** Scatterplot of log_2_ ITC density vs. CYP1B1 histoscore in 80 patients with TNBC. White and black points represent patients with TNBC with RCB class 0/1 and RCB class 2/3 outcomes, respectively.

RCB is a more accurate predictor of long-term risk of recurrence after NAC than classical tumor staging (5). To assess the association of TME metrics and CYP1B1 histoscore with RCB, the RCB class of the pCR− cohort was determined retrospectively. For analysis, RCB classes 0 and 1 were combined as a favorable outcome group and RCB classes 2 and 3 were combined as a poor outcome group. Of our 80-patient cohort, 48 and 32 patients were RCB class 0/1 and 2/3, respectively. As with the pCR+/− response grouping, RCB class was significantly associated with TME metrics and CYP1B1 histoscore. The majority of patients with sTIL density >30% were RCB class 0/1, whereas the majority of patients with sTIL density <30% were RCB class 2/3 (Fig. 4A). Furthermore, the majority of patients with sTIL density >30% showing RCB class 2/3 disease (an outlier subset) had a CYP1B1 histoscore >240 (Fig. 4A). In TME subgroups defined by ITC density as well as sTIL density, 15% of TCI-TNBC, 40% of TCR-TNBC, and 60% of T cell–dessert TNBC were associated with an RCB class 2/3 outcome (Fig. 4B; Table 2). As noted in the pCR+/− response grouping, CYP1B1 histoscore was associated with RCB response group only in TNBC with ITC density <15% (TCE-TNBC; Fig. 4B). The frequency of an RCB class 2/3 outcome in TCE-TNBC was lowest in patients with CYP1B1 histoscore 0 to 60, intermediate in patients with CYP1B1 histoscore 61 to 240, and highest in patients with CYP1B1 histoscore >240 (Table 3).

**Table 2:** RCB class 2/3 outcomes in relation to immunotype in TNBC (*N* = 80)

Immunotype	*N*	RCB 0/1 (*N*)	RCB 2/3 (*N*)	RCB 2/3 (%)	*P*
TCD	25	10	15	60%	<0.01*
TCR	35	21	14	40%	
TCI	20	17	3	15%	

Abbreviations: RCB, residual cancer burden; TCD, T-cell dessert; TCR, T-cell restricted; TCI, T cell infiltrated.

*P < 0.01 and *X*^2^ = 6.94 for the frequency of RCB 2/3 in TCD + TCR TNBC vs. TCI-TNBC. *P* < 0.025 and *X*^2^ = 6.06 for the frequency of RCB 2/3 in TCD-TNBC vs. TCR + TCI TNBC.

**Table 3:** RCB class 2/3 outcomes in relation to CYP1B1 histoscore in TCE-TNBC (*N* = 60)

CYP1B1 histoscore	*N*	RCB 0/1 (*N*)	RCB 2/3 (*N*)	RCB 2/3 (%)	*P*
0–60	30	21	9	30%	<0.01*
61–240	18	8	10	56%	
>240	12	2	10	83%	

*P < 0.01 and *X*^2^ = 7.35 for the frequency of RCB 2/3 in TNBC with CYP1B1 histoscore 0 to 240 vs. TNBC with CYP1B1 histoscore >240. *P* < 0.01 and *X*^2^ = 8.08 for TNBC with CYP1B1 histoscore 0 to 60 vs. TNBC with CYP1B1 histoscore >60.

### Association of elevated CYP1B1 in TNBC with resistance to doxorubicin

To assess the relationships of RCB and CYP1B1 histoscore with specific chemotherapeutics, a retrospective chart review was conducted. Two patients were excluded from this portion of the analysis because they did not complete their planned chemotherapy, and three patients were excluded because they had a history of ipsilateral breast cancer. Two of the patients with recurrent cancer had been previously treated with doxorubicin and the two patients who did not complete chemotherapy had received two cycles of doxorubicin. Interestingly, all five excluded patients had elevated CYP1B1 and RCB class 2/3 disease after NAC. Notably, exclusion of these five patients did not significantly alter the conclusions reached when the remaining patients were analyzed.

Of the 75 patients analyzed, 33 received doxorubicin as part of the initial phase of their NAC regimen. Of these, 32 received concurrent cyclophosphamide. One received concurrent Taxotere. The NAC regimen of 28 patients treated initially with doxorubicin included a second phase; 18 patients received taxane/carboplatin and 10 received a taxane alone. The remaining 42 patients received a taxane as part of their initial chemotherapy, of whom 39 received concurrent carboplatin (TC). Two received concurrent cyclophosphamide. Of the 39 patients treated with TC first, 21 received no additional chemotherapy and 18 were treated in a second phase with doxorubicin and cyclophosphamide. Overall, 78% of patients whose initial regimen was taxane based and 45% of patients whose initial therapy was doxorubicin based were RCB 0/1 at the end of therapy.

When RCB data for the 75-patient cohort were analyzed in relation to the initial form of chemotherapy used, a clear association was observed in patients with elevated CYP1B1 but not in patients with low CYP1B1 (Table 4). These data suggested that the association between elevated CYP1B1 and RCB class 2/3 outcomes observed in this study was primarily because of patients treated initially with doxorubicin and cyclophosphamide. Of note, among 39 patients treated initially with TC, 17 also received pembrolizumab. Importantly, only one of 14 patients with elevated CYP1B1 who received TC as the initial therapy without pembrolizumab had RCB class 2/3 residual disease. This indicated that the better outcome observed with TC as the initial chemotherapy was unrelated to the use of pembrolizumab.

**Table 4:** RCB in TNBC CYP1B1 subgroups in relation to the initial form of chemotherapy

		Initial chemotherapy		
CYP1B1 subgroup	Response subgroup	Doxorubicin	Taxane/platinum	*P*
Low (*H*-score < 75)	RCB 0/1	14	16	ns
	RCB 2/3	6	5	
High (*H*-score > 75)	RCB 0/1	1	17	<0.001
	RCB 2/3	12	4	

Abbreviations: *H*-score, weighted histoscore; ns, not significant.

*P* < 0.001 and *X*^2^ = 17.298 for the frequency of RCB class 0/1 vs. class 2/3 outcome in patients with CYP1B1 >75 who received doxorubicin and cyclophosphamide vs. taxane and platinum salt in the initial phase of neoadjuvant chemotherapy. *P* < 0.001 and *X*^2^ = 12.337 for the frequency of RCB class 0/1 vs. class 2/3 outcome in TNBC with CYP1B1 <75 vs. CYP1B1 >75 for patients treated initially with doxorubicin and cyclophosphamide.

## Discussion

This study investigated a critical unresolved question in TNBC, namely why some patients experience a complete response to NAC while others have significant residual disease at the end of therapy. MS analysis of a discovery cohort identified 72 proteins as candidate biomarkers for chemoresistance including CYP1B1. IHC validated the association of elevated CYP1B1 with RCB class 2/3 residual disease in TCE-TNBC but not in TCI-TNBC. Correlation with chemotherapy regimens suggested that TNBC with elevated CYP1B1 is significantly more resistant to doxorubicin than TNBC with low CYP1B1. The association between chemotherapy resistance and elevated CYP1B1 was not observed in patients treated primarily with a taxane/carboplatin-based regimen.

Early IHC studies demonstrated the expression of CYP1B1 in neoplastic cells of multiple cancer types (36, 37), leading to the suggestion that CYP1B1 is a universal cancer marker (36). Concurrent with these earlier studies, the drug-metabolizing activity of CYP1B1 suggested a possible mechanism for chemotherapy resistance (38). Indeed, knockdown of CYP1B1 was recently shown to enhance the response of TNBC cell lines to paclitaxel, 5-fluorouracil, and cisplatin (39). However, CYP1B1 also activates WNT/β-catenin signaling and epithelial–mesenchymal transition (35) and suppresses apoptosis (40). Furthermore, activation of the aryl hydrocarbon receptor (AhR), a key regulator of CYP1B1 expression, has pleiotropic pro-cancer and anti-inflammatory effects beyond the induction of CYP1B1 (41). Thus, the relationship between CYP1B1 abundance and chemotherapy resistance may be more complex than the detoxification of chemotherapeutic drugs.

CYP1B1 transcription is induced by AhR signaling (42), which in turn is activated by endogenous tryptophan catabolites (43). Thus, our results may implicate tryptophan catabolism as well as AhR activation in TNBC chemoresistance. Tryptophan catabolites also induce effector T-cell apoptosis/dysfunction (44, 45) and promote the differentiation of naïve CD4^+^ T cells into FOXP3^+^ regulatory T cells (46), suggesting a possible link between the upregulation of CYP1B1 and T-cell exclusion in TNBC. The expression of tryptophan-2,3-dioxygenase (TDO2), an enzyme that converts tryptophan into the AhR agonist kynurenine (KYN), correlates with poor survival in TNBC (47). TDO2 is induced by AhR signaling, creating a positive feedback loop (48, 49). Activation of TDO2–KYN–AhR signaling induces epithelial–mesenchymal transition, invasive capacity, and anchorage-independent survival in TNBC cell lines (50). Although speculative, CYP1B1 may be a biomarker for activation of the TDO2–KYN–AhR cycle.

The majority of patients in this study who had CYP1B1^+^ TCE-TNBC and were treated initially with doxorubicin had RCB class 2/3 residual disease at the end of therapy (Table 4). This finding suggests that the assessment of TME and CYP1B1 may identify a subset of patients with TNBC who respond poorly to anthracycline-based chemotherapy. Most patients with CYP1B1^+^ TCE-TNBC treated initially with doxorubicin were also resistant to TC administered during a second phase of their NAC regimen. Together, these findings are consistent with a model proposed earlier (51), in which doxorubicin induces a drug-tolerant state in a subset of patients with TNBC. Doxorubicin activates AhR signaling in the cardiomyocytes of adult rats (52), but its effect on human breast cancer cells is less well studied. Potentially, tumors in which the TDO2–KYN–AhR signaling pathway is activated may respond differentially to exogenous AhR agonists.

Most patients in this study were treated before the approval of immunotherapy for patients with nonmetastatic TNBC. A future study will focus on patients resistant to the Keynote 522 regimen (53), which became the standard NAC regimen for stage II to III TNBC in July 2021 and uses paclitaxel and carboplatin as the initial chemotherapy.

The association between elevated CYP1B1 and resistance to doxorubicin chemotherapy identified in this study should be confirmed by IHC analysis of archival samples from patients treated in a clinical trial setting. If confirmed, this pattern of chemotherapy resistance could guide future clinical trials.

## Supplementary Material

Supplementary Figure S1 Histologic demonstration of targeted in situ enzymatic digestion.

Supplementary Table S1 Tumor cellularity, sTIL density and volume of nanoLC-MS/MS discovery TNBC cohort.

Supplementary Figure S2 Histology of nanoLC-MS/MS discovery TNBC cohort.

Supplementary Table 2 Differentially abundant proteins identified by nanoLC-MS/MS in TNBC with RCB class 0 versus RCB class 2/3 outcomes
