# Supplementary figures and images for: Protein-Level Analysis of Differential Response to Chemotherapy in Triple-Negative Breast Cancer Identifies CYP1B1 as a Biomarker for Chemotherapy Resistance
Source: Cancer Res Commun. 2025 Jul 1;5(7):1060–9. doi: 10.1158/2767-9764.CRC-25-0034 (PMC12210225; doi:10.1158/2767-9764.CRC-25-0034)

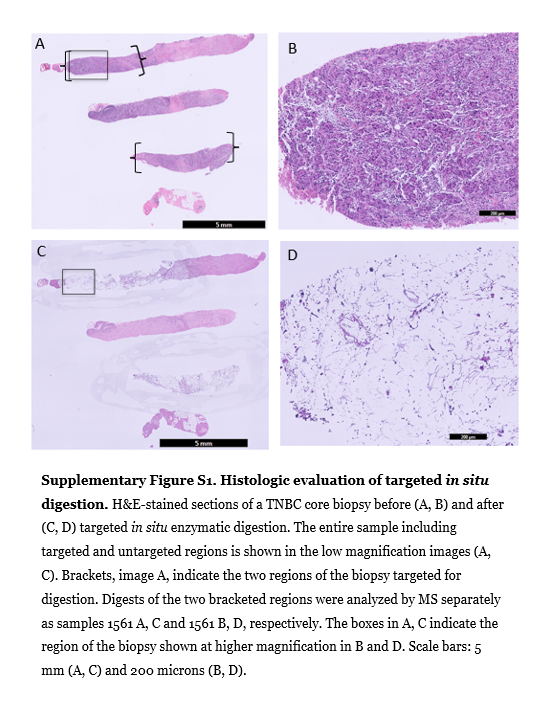

Supplement: Supplementary Figure S1 — Histologic demonstration of targeted in situ enzymatic digestion. [file crc-25-0034_supplementary_figure_s1_suppsf1.png]

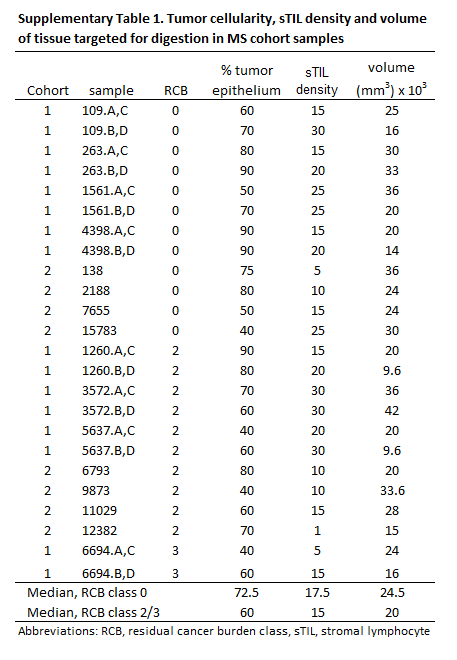

Supplement: Supplementary Table S1 — Tumor cellularity, sTIL density and volume of nanoLC-MS/MS discovery TNBC cohort. [file crc-25-0034_supplementary_table_s1_suppst1.png]

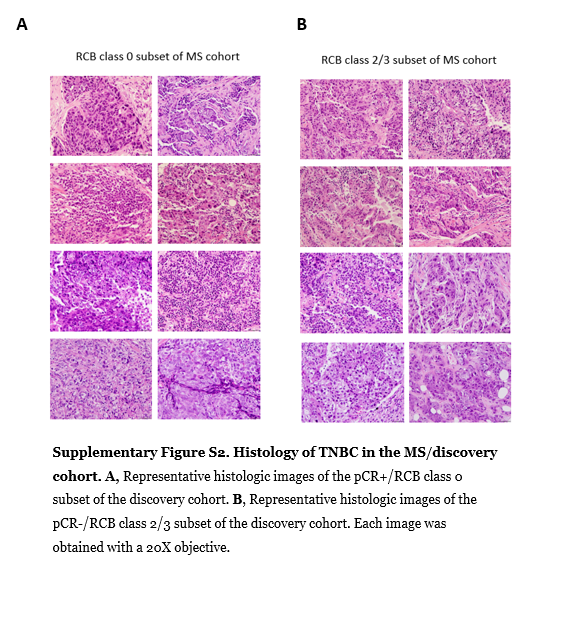

Supplement: Supplementary Figure S2 — Histology of nanoLC-MS/MS discovery TNBC cohort. [file crc-25-0034_supplementary_figure_s2_suppsf2.png]
