# Supplementary material for: Protein-Level Analysis of Differential Response to Chemotherapy in Triple-Negative Breast Cancer Identifies CYP1B1 as a Biomarker for Chemotherapy Resistance
Source: Cancer Res Commun. 2025 Jul 1;5(7):1060–9. doi: 10.1158/2767-9764.CRC-25-0034 (PMC12210225; doi:10.1158/2767-9764.CRC-25-0034)
Supplement: Supplementary Table 2 — Differentially abundant proteins identified by nanoLC-MS/MS in TNBC with RCB class 0 versus RCB class 2/3 outcomes [file crc-25-0034_supplementary_table_2_suppst2.docx]

**Supplementary Table S2. Differentially abundant proteins in TNBC associated with pCR+ and pCR- outcomes (N = 16)**

| **Gene names** | **Protein names** | **Difference [(pCR-)-(pCR+)]** | **-LOG(P-VALUE)** |
| --- | --- | --- | --- |
| ITGA6 | Integrin alpha-6;Integrin alpha-6 heavy chain;Integrin alpha-6 light chain;Processed integrin alpha-6 | -2.544129857 | 3.304573469 |
| ERMP1 | Endoplasmic reticulum metallopeptidase 1 | -2.294436914 | 4.147749784 |
| SORBS2 | Sorbin and SH3 domain-containing protein 2 | -2.242968957 | 4.999232181 |
| DBI | Acyl-CoA-binding protein | -2.038580197 | 5.129329694 |
| TTN | Titin | -1.665185981 | 2.104707259 |
| NDRG2 | Protein NDRG2 | -1.548790216 | 3.820225027 |
| CGN | Cingulin | -1.510648763 | 2.100865838 |
| GAA | Lysosomal alpha-glucosidase;76 kDa lysosomal alpha-glucosidase;70 kDa lysosomal alpha-glucosidase | -1.468131622 | 3.74730858 |
| DDAH2 | Putative hydrolase DDAH2 | -1.419410335 | 4.294537572 |
| AK1 | Adenylate kinase isoenzyme 1 | -1.413685384 | 4.623389961 |
| GLO1 | Lactoylglutathione lyase | -1.300421626 | 5.304040375 |
| PTK7 | Inactive tyrosine-protein kinase 7 | -1.285939058 | 6.217906341 |
| PREP | Prolyl endopeptidase | -1.266847725 | 1.994769616 |
| RMDN1 | Regulator of microtubule dynamics protein 1 | -1.245661338 | 4.94936288 |
| NIBAN2 | Protein Niban 2 | -1.231344382 | 5.740055664 |
| GFUS | GDP-L-fucose synthase | -1.208955005 | 2.924022495 |
| SWAP70 | Switch-associated protein 70 | -1.190290548 | 5.141520992 |
| LEMD2 | LEM domain-containing protein 2 | -1.182021989 | 4.028435079 |
| NIBAN1 | Protein Niban 1 | -1.163925153 | 3.025724547 |
| HMGN1 | Non-histone chromosomal protein HMG-14 | -1.15984439 | 2.574143579 |
| GNA11 | Guanine nucleotide-binding protein subunit alpha-11 | -1.148915397 | 2.249141579 |
| MACROH2A2 | Core histone macro-H2A.2 | -1.137530636 | 3.344124056 |
| CMAS | N-acylneuraminate cytidylyltransferase | -1.127136063 | 3.685519499 |
| LMNA | Prelamin-A/C;Lamin-A/C | -1.122819883 | 5.677246664 |
| KIAA1217 | Sickle tail protein homolog | -1.101086802 | 3.107603206 |
| ALDH6A1 | Methylmalonate-semialdehyde/malonate-semialdehyde dehydrogenase [acylating], mitochondrial | -1.093143543 | 2.695413375 |
| NUMA1 | Nuclear mitotic apparatus protein 1 | -1.089671806 | 3.147750067 |
| GOT1 | Aspartate aminotransferase, cytoplasmic | -1.079163569 | 4.52804241 |
| RAB25 | Ras-related protein Rab-25 | -1.07003053 | 2.162912199 |
| AHCYL1;AHCYL2 | S-adenosylhomocysteine hydrolase-like protein 1;Adenosylhomocysteinase 3 | -1.068612708 | 3.549757791 |
| EMILIN1 | EMILIN-1 | -1.037144025 | 3.929474617 |
| ALDH3A2 | Aldehyde dehydrogenase;Aldehyde dehydrogenase family 3 member A2 | -1.028270633 | 3.107604588 |
| CA2 | Carbonic anhydrase 2 | -1.022597445 | 2.624321594 |
| H1-0 | Histone H1.0;Histone H1.0, N-terminally processed | -1.019038465 | 3.396176869 |
| EML2 | Echinoderm microtubule-associated protein-like 2 | -1.017117615 | 2.13665845 |
| GGCT | Gamma-glutamylcyclotransferase;gamma-glutamylcyclotransferase | -1.003787871 | 2.160676502 |
| PLGRKT | Plasminogen receptor (KT) | -0.991600284 | 2.339697863 |
| PBXIP1 | Pre-B-cell leukemia transcription factor-interacting protein 1 | -0.990312391 | 2.438934206 |
| MYO1D | Unconventional myosin-Id | -0.98910896 | 2.422215369 |
| DYNC1I2 | Cytoplasmic dynein 1 intermediate chain 2 | -0.976259055 | 3.8706103 |
| CRIP2 | Cysteine-rich protein 2 | -0.972223979 | 3.523054549 |
| SUN2 | SUN domain-containing protein 2 | -0.930088909 | 4.809185908 |
| GRHPR | Glyoxylate reductase/hydroxypyruvate reductase | -0.921741221 | 2.240164828 |
| PGM3 | Phosphoacetylglucosamine mutase;phosphoacetylglucosamine mutase | -0.917351105 | 2.311039888 |
| SEPTIN8 | Septin-8;Septin | -0.915389794 | 2.666036975 |
| TRIM28 | Transcription intermediary factor 1-beta | -0.906967499 | 7.229165087 |
| VAPA | Vesicle-associated membrane protein-associated protein A | -0.839706845 | 5.949977968 |
| MYH10 | Myosin-10 | -0.815151797 | 3.049649119 |
| SH3BGRL | Adapter SH3BGRL | -0.805057738 | 3.507697532 |
| ACTN4 | Alpha-actinin-4 | -0.803500546 | 4.406266402 |
| EMD | Emerin | -0.802736247 | 3.865760137 |
| FKBP3 | Peptidyl-prolyl cis-trans isomerase FKBP3 | -0.794650095 | 3.646511181 |
| S100A13 | Protein S100-A13 | -0.791872643 | 2.419029506 |
| PGRMC1 | Membrane-associated progesterone receptor component 1 | -0.75323889 | 2.663485931 |
| S100A11 | Protein S100-A11;Protein S100-A11, N-terminally processed | -0.743998757 | 2.961438518 |
| TPM4 | Tropomyosin alpha-4 chain | -0.672156952 | 3.277254777 |
| ANXA1 | Annexin A1;Annexin Ac2-26;Annexin | -0.660950043 | 2.92244612 |
| TMEM43 | Transmembrane protein 43 | -0.653441694 | 2.935604222 |
| VCL | Vinculin | -0.606821131 | 2.976307788 |
| CORO1C | Coronin-1C;Coronin | -0.581587438 | 4.532602796 |
| RAB14 | Ras-related protein Rab-14 | -0.576516999 | 3.282491391 |
| MDH1 | Malate dehydrogenase, cytoplasmic;Malate dehydrogenase | -0.57585084 | 4.297579933 |
| PFKL | ATP-dependent 6-phosphofructokinase, liver type | -0.529108719 | 3.500260036 |
| RPL28 | Large ribosomal subunit protein eL28;60S ribosomal protein L28 | 0.492753223 | 4.558386959 |
| HSPA4 | Heat shock 70 kDa protein 4 | 0.578068292 | 4.326600528 |
| ATAD3A;ATAD3B | ATPase family AAA domain-containing protein 3A;ATPase family AAA domain-containing protein 3B | 0.644186929 | 3.212944299 |
| SHMT2 | Serine hydroxymethyltransferase, mitochondrial;glycine hydroxymethyltransferase | 0.655054516 | 3.433179348 |
| AP1G1 | AP-1 complex subunit gamma-1 | 0.769390044 | 2.974867978 |
| HSPH1 | Heat shock protein 105 kDa | 0.773353259 | 4.609360254 |
| HSD17B4 | Peroxisomal multifunctional enzyme type 2;(3R)-hydroxyacyl-CoA dehydrogenase;Enoyl-CoA hydratase 2 | 0.783621258 | 3.859688123 |
| TCIRG1 | V-type proton ATPase 116 kDa subunit a 3;V-type proton ATPase subunit a | 0.789463441 | 2.432499181 |
| CDC5L | Cell division cycle 5-like protein | 0.818401116 | 3.371846104 |
| MARS1 | Methionine--tRNA ligase, cytoplasmic | 0.836786588 | 2.375728011 |
| ADSS2 | Adenylosuccinate synthetase isozyme 2 | 0.846231602 | 2.681051138 |
| SEC23IP | SEC23-interacting protein | 0.873181034 | 2.661008851 |
| AIMP1 | Aminoacyl tRNA synthase complex-interacting multifunctional protein 1;Endothelial monocyte-activating polypeptide 2 | 0.874327721 | 2.813436826 |
| LAMTOR1 | Ragulator complex protein LAMTOR1 | 0.893173297 | 2.473083555 |
| TM9SF4 | Transmembrane 9 superfamily member 4;Transmembrane 9 superfamily member | 0.908066626 | 2.603762051 |
| DDX18 | ATP-dependent RNA helicase DDX18 | 0.913590935 | 2.920900137 |
| NDUFB9 | NADH dehydrogenase [ubiquinone] 1 beta subcomplex subunit 9 | 0.914492713 | 3.910244933 |
| RPL32 | Large ribosomal subunit protein eL32 | 0.914527098 | 2.948985071 |
| USP10 | Ubiquitin carboxyl-terminal hydrolase 10 | 0.916938411 | 3.295546098 |
| HNRNPUL2 | Heterogeneous nuclear ribonucleoprotein U-like protein 2 | 0.926249778 | 3.213184652 |
| DDX21 | Nucleolar RNA helicase 2 | 0.946923627 | 3.881234803 |
| FKBP15 | FK506-binding protein 15 | 0.947873778 | 2.75845412 |
| LBR | Delta(14)-sterol reductase LBR | 0.97477474 | 2.215499517 |
| TOP1 | DNA topoisomerase 1 | 0.977451148 | 2.268420911 |
| NSUN2 | RNA cytosine C(5)-methyltransferase NSUN2 | 0.979746218 | 3.056338133 |
| PTPN1 | Tyrosine-protein phosphatase non-receptor type 1;Tyrosine-protein phosphatase non-receptor type | 0.995487496 | 3.057524297 |
| RFC4 | Replication factor C subunit 4 | 1.00354215 | 2.841493761 |
| ALDH2 | Aldehyde dehydrogenase, mitochondrial | 1.020943262 | 2.807670935 |
| SLC25A10 | Mitochondrial dicarboxylate carrier | 1.032494695 | 2.647998444 |
| DRG1 | Developmentally-regulated GTP-binding protein 1 | 1.033293742 | 2.689414908 |
| VTN | Vitronectin;Vitronectin V65 subunit;Vitronectin V10 subunit;Somatomedin-B | 1.045805825 | 2.1013916 |
| SART3 | Squamous cell carcinoma antigen recognized by T-cells 3 | 1.061016145 | 2.781638343 |
| PES1 | Pescadillo homolog | 1.067316682 | 2.380305658 |
| PSME3 | Proteasome activator complex subunit 3 | 1.068266206 | 2.477898465 |
| SF3A1 | Splicing factor 3A subunit 1 | 1.075451727 | 2.510600161 |
| ADSL | Adenylosuccinate lyase | 1.076563464 | 2.769840443 |
| TRIP13 | Pachytene checkpoint protein 2 homolog | 1.094766025 | 2.987751124 |
| BLVRA | Biliverdin reductase A | 1.101480537 | 3.171390642 |
| P4HA1 | Prolyl 4-hydroxylase subunit alpha-1 | 1.116229048 | 2.291178053 |
| BSG | Basigin | 1.117464604 | 2.482030478 |
| FKBP5 | Peptidyl-prolyl cis-trans isomerase FKBP5 | 1.11839985 | 2.435980464 |
| KPNA2 | Importin subunit alpha-1 | 1.129401454 | 2.415874225 |
| EIF2B4 | Translation initiation factor eIF2B subunit delta | 1.149553696 | 2.973339542 |
| PGAM5 | Serine/threonine-protein phosphatase PGAM5, mitochondrial | 1.150752959 | 4.20054332 |
| DNAJA2 | DnaJ homolog subfamily A member 2 | 1.156809745 | 2.545151865 |
| BLMH | Bleomycin hydrolase | 1.161363928 | 2.780907133 |
| NAT10 | RNA cytidine acetyltransferase | 1.188142909 | 3.626896591 |
| OGN | Mimecan | 1.18999996 | 2.399722466 |
| PMPCA | Mitochondrial-processing peptidase subunit alpha | 1.194846489 | 2.05207002 |
| HLA-DQA1 |  | 1.198529279 | 3.147305051 |
| SLC3A2 | Amino acid transporter heavy chain SLC3A2 | 1.206587685 | 5.984910153 |
| ATP5MK | ATP synthase membrane subunit K, mitochondrial | 1.216738021 | 3.527318893 |
| SULT1A1;SULT1A2 | Sulfotransferase 1A1;Sulfotransferase;Sulfotransferase 1A2 | 1.21879688 | 2.161742941 |
| SNRPE | Small nuclear ribonucleoprotein E | 1.221502975 | 2.744689217 |
| BZW2 | eIF5-mimic protein 1 | 1.231149347 | 2.532344649 |
| IPO7 | Importin-7 | 1.232620354 | 3.422057505 |
| GCN1 | Stalled ribosome sensor GCN1 | 1.237137892 | 4.348635693 |
| FARSA | Phenylalanine--tRNA ligase alpha subunit;phenylalanine--tRNA ligase | 1.243416945 | 3.772435599 |
| SLC2A1 | Solute carrier family 2, facilitated glucose transporter member 1 | 1.253839422 | 2.546537674 |
| HSP90AB4P | Putative heat shock protein HSP 90-beta 4 | 1.263130223 | 3.706332017 |
| VCAM1 | Vascular cell adhesion protein 1;Soluble Vascular Cell Adhesion Molecule-1 | 1.272329692 | 3.348791694 |
| SLIRP | SRA stem-loop-interacting RNA-binding protein, mitochondrial | 1.332042579 | 3.554923446 |
| SLC25A22;SLC25A18 | Mitochondrial glutamate carrier 1;Mitochondrial glutamate carrier 2 | 1.355990578 | 5.237884966 |
| H1-3 | Histone H1.3 | 1.363879177 | 1.930012419 |
| PABPC4 | Polyadenylate-binding protein 4;Polyadenylate-binding protein | 1.376986371 | 6.576245547 |
| CHD4 | Chromodomain-helicase-DNA-binding protein 4;DNA helicase | 1.39432606 | 2.339891012 |
| SLC7A5 | Large neutral amino acids transporter small subunit 1 | 1.397043467 | 4.218125065 |
| CSE1L | Exportin-2 | 1.506094844 | 6.17141437 |
| IGKV3-20 | Immunoglobulin kappa variable 3-20 | 1.665961495 | 4.757138027 |
| COL14A1 | Collagen alpha-1(XIV) chain | 1.958993514 | 3.543458174 |
| CYP1B1 | Cytochrome P450 1B1 | 2.200894347 | 2.680809514 |
| HLA-G | HLA class I histocompatibility antigen, alpha chain G;Soluble HLA class I histocompatibility antigen, alpha chain G | 2.508241274 | 4.065778386 |
